# Measurement of age-of-acquisition in morphologically rich languages: Insights from Kannada and Filipino

**DOI:** 10.3758/s13428-025-02876-z

**Published:** 2025-12-01

**Authors:** Katrina May Dulay, Jelena Mirković, Margaret Mary Rosary Carmel Fua, Deeksha Prabhu, Sonali Nag

**Affiliations:** 1 Department of Psychology and Neuroscience, City St George’s, University of London, London, UK; 2 School of Education, Language, and Psychology, York St John University, York, UK; 3 College of Education, University of the Philippines Diliman, Quezon City, Philippines; 4 The Promise Foundation, Bengaluru, India; 5 Department of Education, University of Oxford, 15 Norham Gardens, Oxford, OX2 6PY UK

**Keywords:** Age-of-acquisition, AoA, Child language acquisition, Agglutinative, Morphologically rich

## Abstract

In this study, we present age-of-acquisition (AoA) ratings for 885 Kannada and Filipino words as a new resource for research and education purposes. Beyond this, we consider the methodological and theoretical considerations of measuring AoA in morphologically rich, specifically agglutinative, languages, to study child language acquisition. Parents, teachers, and experts provided subjective ratings of when they thought a child acquired each word. Results were generally consistent between the two languages. Mixed-effects models demonstrated that word characteristics, including parts-of-speech category, word length, and age band of first occurrence in a print corpus, were significantly related to AoA ratings, whereas rater characteristics, including participant type, age, gender, and number of languages spoken, had generally non-significant associations with AoA ratings. The number of morphemes was significantly associated with AoA ratings in some analyses; however, crosslinguistic differences in the directionality of the relationships suggested the need to investigate underlying drivers of morphological complexity such as morpheme frequency, transparency/consistency, and function. The age-of-acquisition ratings were internally reliable and demonstrated consistency with the first occurrences of words in print and known trends in child language research. The results demonstrate the potential of these resources and open new directions for AoA research in morphologically rich languages.

**Supplementary Information:**

The online version contains supplementary material available at 10.3758/s13428-025-02876-z.

## Introduction

Age-of-acquisition (AoA) refers to when a word is first learned. Typically, this is measured through adults’ retrospective recall of when they first learned a particular word (Carroll & White, 1973). The relationship between AoA and lexical processing is well documented in psycholinguistics; that is, words that are learned earlier in life are typically faster to access, name, and recognize than words that are learned later in life (Elsherif et al., 2023). Recently, a number of AoA studies focused on identifying potential linguistic factors that predict children’s word learning in different languages. In parent report studies, age-of-acquisition was estimated primarily using data from the MacArthur-Bates Communicative Development Inventory (MB-CDI: Fenson et al., 1993), a widely used checklist measure in which parents reported whether a child “understood” or “produced” words in a given language. This provides an alternative AoA metric for researchers who are specifically interested in uncovering patterns of child language acquisition.

The current study introduces age-of-acquisition word lists in two morphologically rich languages, Kannada and Filipino, with AoA ratings derived from adults’ estimates of when a child understands a given word. Kannada is a Dravidian language spoken by over 58 million speakers primarily in the Karnataka region of southern India (Eberhard et al., 2023). Filipino is an Austronesian language spoken by 76 million people^1^ in the Philippines (Eberhard et al., 2023). Beyond providing a new set of norms for research, education, and language intervention purposes, the study aims to identify relevant linguistic and demographic characteristics that drive age-of-acquisition estimates and to consider potential implications for child language acquisition in two major Asian languages. Among these characteristics, we consider the unique contributions of morphology, a feature typically overlooked in AoA research (Elsherif et al., 2023), yet a definitive characteristic of the morphologically rich agglutinative languages in the current study. Finally, this study squarely addresses the current underrepresentation of such languages both in AoA (see Łuniewska et al., 2016) and child language research (see Kidd & Garcia, 2022).

## Can age-of-acquisition norms be used to study child language acquisition?

There is interest in how well AoA norms map onto the sequence of child vocabulary development, and for this, the methods used for norm development matter. In adults’ self-rated AoA studies, mean ratings are computed from participants’ responses on a Likert scale representing different age bands or using age estimates in years (Carroll & White, 1973; Kuperman et al., 2012). In parental checklist-based AoA studies, reports are aggregated to estimate when a word is likely to be learned; for example, by identifying the youngest age at which over 50% of children could produce a word or through model estimation methods (Braginsky et al., 2019; Fourtassi et al., 2020; Portelance et al., 2023). Two studies utilized direct measures of children’s AoA. Brysbaert and Biemiller (2017) sought to validate test-based AoA norms based on tests of children’s English word knowledge across primary and secondary grade levels. Each word was assigned to a grade level at which 50% or more children knew its meaning. Moreover, Smolík and Filip (2022) introduced a novel AoA measure of the age of earliest occurrence of words in a child speech corpus based on recordings when children were around 2 to 3 years old.

Both adults’ self-rated AoA and parent checklist-based AoA have moderate-to-strong correlations with direct tests of children’s vocabulary knowledge, providing support for the validity of these measures (Heilmann et al., 2005; Łuniewska et al., 2016). For example, adult self-rated AoA measures had strong correlations (*r* = *.*76) with the Brysbaert and Biemiller (2017) test-based AoA norms. However, evidence across 25 languages reported only weak to moderate mean correlations between adults’ self-rated and parent checklist-based AoA (*r* = –.43 for understanding and *r* = *– *.39 for production; Łuniewska et al., 2016), leading Portelance et al. (2023) to argue that these methods, while similar in intent, are not the same. Checklist-based estimations typically measure vocabulary in the earliest stages of vocabulary development (e.g., between ages of 8 and 36 months), whereas adults’ self-rated AoA studies typically include words learned across childhood and adulthood.

To unpick the relationships among different types of AoA measures, Smolík and Filip (2022) focused on data in English and compared the relationships among adult self-rated AoA, parent checklist-based AoA, test-based AoA norms, and their novel corpus-based AoA measure. The patterns of correlations among these measures revealed: (1) stronger relationships between parent checklist-based and child speech corpus AoA than with other measures, (2) stronger relationships between adult self-rated AoA and test-based AoA than with other measures, and (3) no relationship between child speech corpus and test-based AoA measures. Results also demonstrated that the adult self-rated AoA equivalents of words found in the child speech corpus tended to identify the words as later acquired. Taken together, the authors suggested that parent checklist-based and corpus-based AoA measures better captured word acquisition at earlier ages; whereas, adult self-rated AoA and test-based AoA measures better represented acquisition during school age.

When comparing self-rated AoA with children’s vocabulary performance using data from English, Kuperman et al. (2012) observed that AoA raters underestimated vocabulary growth between 2 and 5 years and overestimated words learned after 9 years. This converges with results from a Norwegian sample which demonstrated that subjective ratings of one’s own learning underestimated the number of words that children knew at age 3 (Lind et al., 2015). However, while parental checklists such as the MB-CDI provide better estimates of vocabulary knowledge in the early years, the item list is constrained to words within the measure and does not correlate well with school-aged children’s vocabulary knowledge (Smolík & Filip, 2022).

Adult AoA measures provide an opportunity to expand the range of word items included, yet researchers have addressed their potential shortcomings around the estimates for younger ages in two ways (e.g., Łuniewska et al., 2016). The first is to recruit participants with recent or ongoing interactions with children. Łuniewska et al. (2016) suggested that parents tended to provide earlier AoA estimates than non-parents. Similarly, Barrow et al. (2019) assessed the validity of Swedish-speaking adults’ self-rated AoA ratings against AoA derived from MB-CDI parent checklists. All except one participant who passed the pre-set validity criterion had children or worked with children, leading them to conclude that self-rated AoA ratings should be provided by participants who are highly familiar with children. A second solution is to modify task instructions from the dominant self-rating format (“When did you learn this word?”) to a child-focused format (“When do children learn this word?”). Results from a Polish subsample in Łuniewska et al. (2016) revealed that adults’ ratings of their own word learning produced later AoA ratings compared to the modified instruction. In the current study, we applied both suggested solutions, and we recruited parents, teachers, and experts in fields related to child language development, and modified the task instruction to focus on children’s word learning up to age 10. Furthermore, we explored potential variations in AoA ratings across participant types given that parents’, teachers’, and experts’ knowledge of child language may differ due to the nature of their experience or training.

## What are the linguistic and demographic factors that drive variation in AoA ratings?

Regardless of the method of measuring AoA, studies note several systematic trends in the sequence of word acquisition, including remarkable crosslinguistic similarities in which words are learned earlier and what predicts AoA ratings. Using adult self-ratings, Łuniewska et al., (2016, 2019) reported moderate to strong correlations in the AoA given to the same 299 nouns and verbs across 32 languages, suggesting that similar learning sequences could apply crosslinguistically. Based on MB-CDI parent checklists across languages, early words tend to comprise names of caregivers (e.g., mommy, daddy), common nouns (e.g., bottle), and everyday expressions (e.g., hi, bye) (Frank et al., 2017; Tardif et al., 2008).

Moreover, word length, indexed by the number of syllables, phonemes, or letters, is similarly known to increase age-of-acquisition across languages, including agglutinative, whether measured through adult self-ratings (e.g., Turkish: Raman et al., 2014; Göz et al., 2017; Japanese: Nishimoto et al., 2005, 2012; Malay and Western Armenian: Łuniewska et al., 2019) or parent checklists (Turkish: Braginsky et al., 2019; Fourtassi et al., 2020). Braginsky et al. (2019) also found that several other factors significantly predicted parent checklist-based AoA in most, if not all, 20 languages studied, including frequency, valence, arousal, concreteness, association of a word with infants, and occurrence within shorter utterances or as the sole or last utterance in child-directed speech.

In adult self-rated AoA research, nouns are the only or most dominant word class included in most studies (see Łuniewska et al., 2016 for a discussion), precluding an analysis of variations in AoA across different parts of speech. In contrast, extensive research on the “noun bias” in early language development suggests that children may learn nouns more easily than verbs in their early vocabulary. Studies involving English-, Mandarin-, and Japanese-speaking children and caregivers reported mixed results, suggesting that the strength of the noun bias may depend on the language used, interaction context, and caregiver input (Ogura et al., 2006; Setoh et al., 2021; Tardif et al., 1999). There is additional cross-linguistic evidence suggesting that varied learning trajectories across parts of speech could be driven by the psycholinguistic properties of the words being learned. The 20-language analysis of MB-CDI parent checklists by Braginsky et al. (2019) suggested different predictors for learning nouns, predicates (verbs, adjectives, and adverbs) and function words (pronouns, prepositions, question words, quantifiers, articles, auxiliary verbs, and connectives). Nouns, verbs, adjectives, and adverbs were more likely to be known by children when they were more frequent and concrete, whereas function words were more likely to be known when they were shorter and occurred in shorter sentences.

To complement the work using parent checklists and in contrast to adult self-rated AoA studies, which focused primarily on nouns, in the current study, we included nouns, predicates (verbs, adjectives, adverbs), and one type of function word (pronouns) in the word list, consistent with studies exploring the noun bias and parts-of-speech variations in child language development.

Finally, mixed trends have been noted for studies investigating the link between the demographic characteristics of AoA study participants, such as education level, gender, age, and bi-/multi-lingual language status (Bird et al., 2001; Kuperman et al., 2012; Łuniewska et al., 2016), and ratings they provide. No significant associations were found between participants’ education level and adult self-rated AoA ratings (Kuperman et al., 2012; Łuniewska et al., 2016). In terms of gender and age, Kuperman et al. (2012) found that women and older participants gave later AoA ratings than men and younger participants, whereas Łuniewska et al. (2016) did not find significant associations between these variables. Bird et al. (2001) suggested that generational differences in vocabulary use could account for differences in AoA ratings between younger and older adults, at least for a small subset of words that reflected technological or lifestyle changes over the years. Finally, Łuniewska et al. (2016) compared monolingual and bi-/multi-lingual raters and found that bi-/multi-linguals provided later AoA ratings than monolinguals. Given the possibility that demographic differences could influence AoA ratings and any inferences we may draw regarding the sequence of word acquisition, as well as the small number of studies available and inconsistent results reported, we examined the potential links between AoA ratings and participants’ education level, gender, age, and language status in this new sample of Kannada and Filipino speakers.

## The case for morphology: What agglutinative languages can contribute to age-of-acquisition and child language research

As Elsherif et al. (2023) noted in their review of age-of-acquisition research, many AoA studies focus on monomorphemic words, and research that considers relationships between AoA and morphologically complex words is relatively limited. In a study that considered inflectional morphology in English, Kuperman et al. (2012) found that the AoA of English base words significantly predicted lexical decision speeds for their inflected forms (e.g., play–played). Drawing from these results, Davies et al. (2016) inferred that the AoA of derived word forms could be related to the AoA of their base forms. Nevertheless, they demonstrated in their own study involving English and Spanish words that morphologically complex forms received later AoA ratings than their corresponding base forms. This occurred whether or not the phonological and semantic relationship between base and complex forms was transparent (e.g., friend–friendly; worth–worthless) or more opaque to derive (e.g., sign–signal; event–eventual).

The two aforementioned studies involve languages that are morphologically simpler than the agglutinative languages in the current study. They rely on binary classifications of morphological complexity (base vs. inflected/derived forms). An assumption behind these categorical classifications is a specific perspective on morphological representations and processing that views morphemes as discrete units (decompositional view; Stevens & Plaut, 2022). This perspective has been contrasted with distributed models of morphology that view morphological representations as graded regularities in the mapping between form and meaning (e.g., Seidenberg & Plaut, 2014; Stevens & Plaut, 2022). There is evidence that distributed models better capture morphological processing in morphologically complex languages, both in adult word processing and in language acquisition (e.g., Engelmann et al., 2019; Mirković et al., 2011). Distributed models emphasize the role of factors such as frequency of lexical and sublexical regularities, and semantic and phonological similarity among morphologically related words (e.g., Gonnerman et al., 2007).

Similar to AoA and lexical processing research in adults, theories of morphological acquisition fall along the continuum from rule-based to distributed views, with the former proposing formal linguistic rules and discrete morphemic representations that govern morphological processes, and the latter assuming that morphologically complex forms emerge through learning the mapping between form and meaning (e.g., Granlund et al., 2019; Seidenberg & Plaut, 2014). Regardless of the theoretical view adopted across studies, young children learning agglutinative languages have shown a sensitivity to different features of morphologically complex words. For example, children as young as one year old demonstrated sensitivity to frequent suffixes in Japanese (Murasugi, 2014) and Hungarian (Ladányi et al., 2020), whereas 3- to 5-year-olds made more accurate productions of inflected forms in Finnish (Engelmann et al., 2019) and Estonian (Granlund et al., 2019) when the forms occurred more frequently in child-directed speech corpora or were part of a larger word neighborhood that shared commonalities in terms of base and inflected forms (phonological neighborhood density). These studies demonstrate the role of both morphological structure and phonological and other sublexical properties in the acquisition of morphologically complex words.

The current study aims to explore the role of morphological complexity in AoA ratings in agglutinative languages. In these languages, words are built by combining morphemes (e.g., adding affixes to root words) in a linear, additive manner, where each morpheme typically has a distinct grammatical or semantic function. Binary classifications of morphological complexity into base vs. morphologically complex forms may not be appropriate when studying these types of languages, as morphologically complex forms are the norm, and bare (base/root) forms occur significantly less frequently than in morphologically simpler languages.

Aro (2017) provides a useful illustration of agglutinative morphological processes in Finnish. Nouns, verbs, adjectives, and pronouns can be inflected to express grammatical relations (e.g., case for nouns, and tense, mood, and person for verbs), as in the case of *istuisimme* (we would sit). Here, the verb *istua* (to sit) has two suffixes attached: *mme* (we) and *isi* (would). Root morphemes can be compounded to form the word *mustaviinimarjamehupullo* (a bottle of black currant juice), comprised of *musta* (black) + *viini* (wine) + *marja* (berry) + *mehu* (juice) + and *pullo* (bottle). Suffixes are also used for derivation (e.g., *kirja* – book, *kirjain*—letter of the alphabet, *kirjasto* – library). A similar reliance on suffixes for inflectional and derivational morphology characterizes Turkish (Durgunoğlu, 2017) and Hungarian (Gervain, 2022; Kiefer & Komlósy, 2011). Across the three languages, authors noted that the variety of inflections available can result in hundreds to thousands of possible word forms across parts-of-speech categories (Aro, 2017; Durgunoğlu, 2017; Gervain, 2022).

More specific to our two languages of interest, Kannada is similarly rich in suffixation for inflectional and derivational morphology. Nouns and pronouns can be marked for person, number, and gender, and verbs can be marked for person, number, and gender agreement with the subject as well as tense, aspect, and mood (Sridhar, 1990; Nag, 2025). In the sentence *huDuganige hedarike aayitu* (The boy was frightened), *huDuga* (boy) is followed by the suffix *-nige*, which is a dative marker that indicates that the boy is the recipient of the action, *hedarike* means ‘fear’, and *aayitu* comprises the root *aagu* (to become) and the suffix -*itu,* which indicates past tense, and has undergone vowel assimilation*.*

In contrast to the suffix-heavy languages discussed, Filipino morphology is notable for the rich use of affixes in a variety of linear positions (prefixes, suffixes, infixes, and circumfixes) (Schachter & Otanes, 1972). Filipino nouns are typically not inflected for case, number, or gender, similar to Japanese (Koda, 2017). However, inflectional verb morphology in Filipino is complex: affixation and reduplication are used to indicate grammatical relations such as focus (which expresses the role of the subject of a sentence) and aspect (which indicates how an action takes place over time). For example, the verb *kumakain* (eating) has the root *kain* (eat) and has a reduplicated C1V1 syllable *ka* and infix *-um-*, which denotes an imperfective aspect (or that the action of eating is ongoing). The infix -*um-* also indicates that when used in a sentence (e.g., *Kumakain si Linda*; Linda is eating), the subject (Linda) is the actor or doer of the action.

As described earlier, a simple binary measure distinguishing root/base and morphologically complex forms as in previous AoA research is unlikely to capture morphological complexity appropriately in agglutinative languages. Additionally, such a measure assumes a particular view of morphological representations and processing. Given this, we opted for a measure that might capture the complexity of agglutinative languages more appropriately than a binary measure, and that is, at the same time, a more theoretically neutral measure: the number of morphemes. This simple measure of morphological complexity was used as a first step in exploring its role in AoA ratings. Based on previous research (e.g., Davies et al., 2016), we might expect that in the current study, words with more morphemes might garner later AoA ratings than those with fewer.

The morphological processes that form multimorphemic words in agglutinative languages inevitably result in increased word length, as expressed in the number of syllables or phonemes. This apparent confound between the number of morphemes and word length may be even more salient and harder to control in agglutinative languages, where every word could comprise multiple possible morphemes. However, to our knowledge, no study has investigated the relationships between the number of morphemes and AoA ratings, and between the number of morphemes and word length, in agglutinative languages. We will examine these relationships in this study.

## Assessing the breadth and reliability of new AoA norms

New age-of-acquisition word lists are typically drawn from existing resources such as language corpora, vocabulary lists, and object names elicited from picture naming tasks, and their validity is established by correlating the new ratings with existing adult-rated AoA norms, equivalent lists in other languages, adult lexical decision times, or developmental norms for vocabulary acquisition (e.g., Brysbaert et al., 2014; Kuperman et al., 2012; Łuniewska et al., 2016). In the current study, Kannada and Filipino words were sourced from two similarly constructed corpora of child-directed print for the TalkTogether project (https://talktogether.web.ox.ac.uk). The corpora comprised award-winning, bestselling, or librarian- and expert-recommended books that contained connected text and were aimed at children between 3 and 10 years old. To maintain consistent criteria for age ratings across publishers, the books received new expert ratings of their suitability for children aged 3–5, 6–8, and 9–10 based on a qualitative evaluation of content, organization of ideas, language, and illustrations and design using the TalkTogether Book Leveling Tool (Padilla et al., 2021). The book ratings enabled tagging individual words by the age band of their first occurrence in the print corpus, providing an estimate of when a child might encounter the word during a shared or independent reading session. This is aligned with statistical learning accounts of language learning and development, which propose that children’s vocabulary is shaped by many varied encounters with words from speech and print, as well as the social interactions that enable these encounters (see Erickson & Thiessen, 2015 for a review). However, one potential limitation of the TalkTogether Book Leveling metric is the lack of a book age rating for children below 3, which is indicative of the types of books selected for the corpus and raises the possibility that books that might otherwise have received a ‘below 3’ rating are judged as appropriate for children aged 3–5 instead. Nevertheless, these ratings provide a level of differentiation between words that are potentially encountered earlier than later in print. Given the absence of typical reference criteria for comparing our age-of-acquisition word lists, we examined the consistency between adult-rated AoA and age of occurrence in print to establish the validity of the new ratings.

Given observed similarities in word acquisition across languages, we may also assess the consistency of the new AoA norms against known trends in children’s early vocabulary from self-rated AoA and parent checklist data in other languages, as well as studies reporting child speech data in Kannada and Filipino. To do this, we examined ten words that attained the earliest AoA ratings in the Kannada and Filipino word lists and compared them against words reported in previously published studies.

## The current study

The current study contributes to the linguistic diversity of age-of-acquisition and child language research by introducing age-of-acquisition ratings in two agglutinative languages, Kannada and Filipino. A focus on agglutinative languages is relevant as they possess linguistic features that are not normally highlighted in AoA research. In particular, we investigate the relevance of morphological complexity (measured by the number of morphemes), its resulting impacts on word length, and its implications for AoA. We have two specific aims: First, we examine how word and rater characteristics might influence age-of-acquisition ratings in Kannada and Filipino. Word characteristics include parts-of-speech category, number of syllables, number of phonemes, number of morphemes, and the age band of first occurrence in print (3–5, 6–8, or 9–10 years). Rater characteristics include participant type (parent, teacher, or expert), age, gender, education level, and number of languages spoken. Our second aim is to introduce new word lists that can be used in child language acquisition research and future experiments, assessments, teaching curricula, and interventions in these two languages. For this, the current study implements methodological improvements on more typical AoA research practices by: a. adapting the task instruction from a rating of one’s own acquisition to a child’s acquisition by experienced raters; b. sourcing words from a children’s print corpus; c. comparing AoA ratings with a new reference criterion for the age band of first occurrence in a children’s print corpus and known trends in children’s early vocabulary in other languages, and d. representing words across different parts of speech apart from nouns. These adaptations aim to improve the breadth and reliability of AoA ratings.

## Method

### Participants

Kannada-speaking (*n* = 74) and Filipino-speaking (*n* = 70) adults were recruited to provide age-of-acquisition (when a word is first learned) ratings for 885 words. Three types of participants comprised the sample: parents, teachers, and experts, and satisfied the following inclusion criteria: (1) must self-identify as a native speaker or very fluent in the target languages (Kannada and Filipino), (2) are parents/caregivers or have recent experience working with typically developing children, and (3) have the means to communicate with the research team and access an online survey. In both study sites (India and the Philippines), participants were recruited purposively. Parent and teacher recruitment was facilitated through non-government organizations and team members with extensive links to local communities and teachers. In the Philippines, parent recruitment focused on two communities with primarily low-income profiles; in India, recruitment was across multiple communities with varying socio-economic statuses. In both countries, research team members approached experts in their immediate networks, who were practitioners, academics/researchers, teacher trainers, or individuals fulfilling a combination of the aforementioned roles.

#### Inclusion criteria for parents

Participants recruited as parents had a child who was at least 10 years old or older and spoke with the parent in the target language. This criterion was decided as pilot testing revealed difficulties among parents with only young children to rate words for older children. The child age criterion reflected the latest age band for the age-of-acquisition survey (age 10 +); hence, the parents are assumed to have experienced children’s language progression within the first 10 years of life.

#### Inclusion criteria for teachers

Teachers were required to have at least 12 months of experience teaching children between preschool to grade 12 using the target language as a medium of instruction. In both countries, these grade levels were attended by children between 3 and 17 years old. Teacher experience included classroom teaching and curriculum development for multiple grade levels.

#### Inclusion criteria for experts

Experts came from fields with knowledge and experience of child language development, such as speech-language therapy, psychology, education, developmental pediatrics, and linguistics. During recruitment, participants were asked to confirm that they had at least 12 months of experience in their field. Additionally, experts should have worked with typically developing children alongside any experience with neurodivergent children or children with special educational needs.

#### Analytic sample

The data from two Filipino participants were excluded from the analyses due to a disclosure that they spoke with their children in English, and an outlier response set wherein 87% of words received the latest age rating. The final analytic samples were predominantly female (Kannada: 84%; Filipino: 87%), with mean ages of *M* = 37.8 years, *SD* = 8.40 (Kannada) and *M* = 40.3 years, *SD* = 9.4 (Filipino).

The Kannada sample consisted of 30% parents, 43% teachers, 27% experts, and the Filipino sample had 53% parents, 29% teachers, and 18% experts. Most participants in the Kannada sample had undergraduate degrees or higher (95%). Due to the purposive recruitment of parent participants from low-income communities, the Filipino sample had a smaller percentage of participants with undergraduate degrees or higher (66%) at the time of the study.

Kannada-speaking parents had either one or two children (*M*_age_ of under 18 s = 11.03 years, 5 years old to 17 years old), whereas Filipino-speaking parents had up to 6 children (*M*_age_ of under 18 s = 13.51 years, 9 months old to 17 years old). Teachers in both samples had an average of 12 years of teaching experience and taught levels between preschool to grade 12 (normative ages between 3 and 17 years old). Most teachers reported having taught at multiple grade levels. Kannada-speaking experts had an average of 7 years of experience, whereas Filipino-speaking experts had an average of 21 years. In the two samples, experts generally identified their field of expertise as speech and language, early childhood education, language/literacy education, and psychology. All had confirmed experience working with typically developing children.

The majority of participants confirmed speaking the target language of the study every day, except two Kannada speakers and one Filipino speaker who spoke the language several times a week. Kannada-speaking participants spoke an average of 3.4 languages (*SD* = 1.1; 3% monolingual, 18% bilingual, 34% trilingual, 28% spoke four languages, and 18% spoke five languages) and 78% of Filipino-speaking participants spoke an average of two languages (*M* = 2.07, *SD* = 0.7; 22% monolingual, 50% bilingual, 27% trilingual, and one participant spoke four languages). English, Hindi, and/or regional languages (e.g., Tulu, Konkani, Telugu) were additionally spoken by participants in the Kannada sample, whereas English and regional languages (e.g., Ilokano, Bisaya, Ilonggo) were also used by participants in the Filipino sample. This level of multilingualism is expected in India and the Philippines: both countries have hundreds of native languages and include English as a medium of instruction (Gonzalez, 1998; Upadhyay & Hasnain, 2017).

### Stimuli

Two lists of 885 words were drawn primarily from the TalkTogether Kannada Child-Directed Print Corpus and TalkTogether Filipino Child-Directed Print Corpus. As described in the Introduction, books in the children’s print corpora were aimed at 3- to 10-year-olds. The AoA word lists (Kannada: https://osf.io/g4dms; Filipino: https://osf.io/j42g7) and technical report (https://osf.io/gnjmr) detailing the word selection process are available at the TalkTogether Open Science Framework repository. The Kannada and Filipino word lists included five parts of speech (POS): nouns, verbs, adjectives, adverbs, and pronouns. Item selection reflected parts-of-speech distributions in the source corpora and presented a larger diversity of POS than many age-of-acquisition studies. Hence, the lists featured more nouns (target: 400) than verbs (target: 275), and more nouns and verbs than the other three POS (target: 70 each). The lists were designed to achieve parity in numbers and general selection methodology, and not for semantic equivalence between languages.

#### Word selection and tagging process

In the early stages of word selection, candidate words were extracted as they appeared in the corpora. The unconstrained list contained diverse word forms, reflecting the morphological richness of Kannada and Filipino. These may include root/base forms, affixed and/or reduplicated forms, and compounds.

Using this approach, the initial Kannada word list consisted solely of multimorphemic words. However, pilot-test participants reported confusion when rating inflected words, especially nouns, verbs, and adjectives, due to the wide variety of forms available. The final modified word list had restrictions on variability and prioritized forms that were considered more common in child-directed speech in Kannada. Thus, some words were presented in citation form: nouns were presented in nominative singular and nominative plural forms, verbs were presented in present tense with variable gender and person markers, and adjectives were presented in the nominative singular form. Adverbs and pronouns remained as they appeared in the corpus. The final Kannada list contained more multimorphemic (72%) than monomorphemic words (28%), consistent with the extensive use of case, number, and gender markers in nouns and pronouns, person, number, gender, tense, and mood markers in verbs, as well as case markers and postpositions in adjectives and adverbs (Nag, 2025; Sridhar, 1990; Tiwari et al., 2017). Three nouns in the Kannada word list were sourced from existing child language and literacy assessments and had no corresponding occurrences in the print corpus; thus, these words were excluded from the analytic dataset.

The final Filipino word list contained similar proportions of monomorphemic (48%) and multimorphemic (52%) words. Fewer modifications were made, as most nouns, pronouns, and adverbs were monomorphemic (64–82%). This reflects the lack of person, number, and gender markers in nouns and the lack of gender-marked pronouns in Filipino (Schachter & Reid, 2018). Furthermore, we excluded all adverbs of manner from the Filipino list as they were indistinguishable from adjectives when read outside of a sentential context. However, to achieve a level of parity with plural nouns in Kannada (suffix -*gaLu*), 9% of Filipino nouns were additionally marked with the syntactic marker *mga*, which functions as a pluralizer. In contrast, 59% of adjectives and 92% of verbs were multimorphemic, as affixes were frequently needed to create these word categories in Filipino. These remained as they appeared in the corpus.

The words were tagged for POS, the number of syllables, phonemes, and morphemes, and the print age band at which a word first occurred in the print corpus. Automated analyzer tools were used to determine the number of phonemes in Kannada (Agrawal & Nag, 2021) and the number of syllables and phonemes in Filipino (Lucasan et al., 2019). In both languages, at least two native-speaking psycholinguistics-trained researchers attached the word tags for POS, syllables (Kannada only), morphemes, and print age band. All automated and manual word tags were reviewed and corrected by senior linguists in the team.

#### Number of syllables, phonemes, and morphemes by parts of speech

Table 1 reports the properties of the words included in the final lists for the number of syllables, phonemes, morphemes, and print age band across the five POS. Both Kannada and Filipino word lists featured multisyllabic words, with 2–3 syllables on average, as well as multimorphemic verbs, with two morphemes on average.

**Table 1:** Word distribution and properties in Kannada and Filipino by parts of speech

Parts of speech	Kannada					Filipino				
	*n*	No. of syllables^a^	No. of phonemes^a^	No. of morphemes^a^	Print age band^b^	*n*	No. of syllables^a^	No. of phonemes^a^	No. of morphemes^a^	Print age band^b^
Noun	400^c^	3.37 *(0.85)*	7.14 *(1.84)*	1.84 *(0.41)*	1.75 *(0.74)*	387	3.07 *(1.07)*	7.03 *(2.35)*	1.39 *(0.65)*	1.64 *(0.71)*
Verb	275	3.49 *(1.15)*	7.75 *(2.74)*	2.19 *(1.14)*	1.63 *(0.66)*	275	3.63 *(0.93)*	8.22 *(2.12)*	2.32 *(0.69)*	1.66 *(0.69)*
Adjective	70	3.23 *(0.82)*	6.93 *(1.64)*	1.79 *(0.48)*	1.70 *(0.77)*	106	3.08 *(0.90)*	6.94 *(1.98)*	1.66 *(0.62)*	1.53 *(0.68)*
Adverb	70	3.87 *(1.20)*	8.31 *(2.59)*	1.83* (0.88)*	1.57 *(0.71)*	44	2.98 *(1.00)*	6.43 *(2.35)*	1.48 *(0.73)*	1.66 *(0.75)*
Pronoun	70	3.31 *(1.07)*	6.74 *(2.45)*	2.01 *(0.79)*	1.39* (0.67)*	73	2.29 *(0.61)*	4.85 *(1.48)*	1.18 *(0.39)*	1.89 *(0.86)*
All words	885	3.43 *(1.01)*	7.37 *(2.29)*	1.96 *(0.80)*	1.67 *(0.72)*	885	3.18 *(1.04)*	7.18 *(2.35)*	1.70 *(0.78)*	1.65 *(0.72)*
Range	-	1–8	2–18	1–6	1–3	-	1–7	2–16	1–4	1–3

^a^Mean number of syllables, phonemes, and morphemes with the standard deviation in italics and parentheses

^b^Print age band (of the word as it appears in the AoA word list) was converted into numeric values wherein 1 = 3 to 5 years, 2 = 6 to 8 years, 3 = 9 to 10 years to compute a mean with the standard deviation in italics and parentheses

^c^Three nouns from the Kannada list were drawn from existing child language and literacy assessments and were not found in the Kannada print corpus. Hence, the mean print age band is computed from 397 Kannada nouns

### Procedure

Participants were asked the Kannada or Filipino equivalent of the question: “At what age do you think a child understands the following words?”. Participants read each word on the list and selected one of seven choices: 0–1 years, 2–3 years, 4–5 years, 6–7 years, 8–9 years, 10 + years, and I don’t know. Participants were encouraged to rate the words in the form in which they appeared.

Due to COVID-19 restrictions at the time of the study, data collection was conducted remotely via Qualtrics. To accommodate different levels of familiarity with digital technologies and survey studies, the research teams in India and the Philippines arranged sessions wherein each participant answered the survey while connected with a research assistant via a voice or video call.

During the initial practice session, the research assistants briefed participants about the survey, asked them to answer a demographic survey, as well as rate the practice lists (see details below). After this, participants rated the main survey lists in four separate sessions, lasting around 40 min each. Each participant provided a total of 1000 ratings, which included 885 AoA ratings and 115 imageability ratings (not reported in this paper). Based on pilot testing, the presentation of stimuli was carefully arranged to reduce participant fatigue effects, order effects, and cognitive load. In each session, participants provided 250 ratings each, with no more than three parts-of-speech and/or type of rating task within each list. Table 2 provides a summary of the stimuli presented across sessions. The order of the four sessions was counterbalanced across participants. Each list had 25–29 blocks, and each block typically contained 7–11 words from the same POS and were either all monomorphemic or all multimorphemic forms. Word presentation order was randomized at different levels: between POS (in the case of sessions with multiple POS), between blocks (sharing the same POS), and within each 7–11 word block.

**Table 2:** Composition of the word lists by session and rating task in Kannada and Filipino

Parts of speech	Kannada					Filipino				
	*n*	List 1	List 2	List 3	List 4	*n*	List 1	List 2	List 3	List 4
Noun	400	250	-	-	150	387	250	-	-	137
Verb	275	-	135	140	-	275	-	91	71	113
Adjective	70	-	-	70	-	106	-	-	106	-
Adverb	70	-	-	-	70	44	-	44	-	-
Pronoun	70	-	-	40	30	73	-	-	73	-
*AoA Total*	885	250	135	250	250	885	250	135	250	250
*Imageability total*^*a*^	115	-	115	-	-	115	-	115	-	-
Total ratings	1000	250	250	250	250	1000	250	250	250	250

*Note.* ^a^ Imageability ratings are not reported in this paper

The practice lists were designed to ease participants into the rating task and to enable a test–retest reliability check. A list of 50 words was drawn from the 885-word pool in each language, with 30 nouns, ten verbs, and ten adjectives selected across print age levels and according to mono/multimorphemic status, with proportions that generally reflected the distribution of the main survey list. The practice list followed the same presentation protocol as the main survey list.

The research assistant and participant agreed on the session schedule for the research assistant to provide study and technical assistance, if needed. On average, Kannada participants completed the four-session survey over ten days (*M* = 9.8, *SD* = 8.9), with a range between same-day completion to 39 days. Filipino participants completed the survey over 3 days (*M* = 2.7 days, *SD* = 3.0), with a range between same-day completions (32%) and up to 14 days.

## Results

The Results section is structured as follows: we first present descriptive statistics of the AoA ratings in the two languages, and then we present the analyses assessing the role of word- and participant-level factors in AoA ratings in each language. The dataset and analysis code used to generate the results are available at the TalkTogether Open Science Framework repository: https://osf.io/ugx4y/?view_only=bd9cb53faefe4fdc93070754f15a70c3.

### Descriptive results

A total of 65,490 and 60,180 ratings were gathered from the Kannada and Filipino samples, respectively. “I don’t know” ratings were given 474 times in Kannada and 685 times in Filipino, representing 0.01% of ratings in each sample. After excluding “I don’t know” ratings and another 222 ratings for three Kannada words with no print corpus occurrences, the resulting analysis was based on a final set of 64,805 ratings in Kannada (from 74 raters) and 59,495 ratings in Filipino (from 68 raters). Table 3 contains summary statistics for AoA by parts of speech in both languages.

**Table 3:** Age-of-acquisition ratings by parts of speech in Kannada and Filipino

Parts of speech	Kannada	Filipino
	Mean AoA^a^	Mean AoA^a^
Noun	3.58* (0.81)*	3.74 *(0.90)*
Verb	3.85 *(0.75)*	3.86 *(0.77)*
Adjective	3.84 *(0.73)*	3.90 *(0.80)*
Adverb	4.03* (0.65)*	3.67 *(0.83)*
Pronoun	3.35 *(0.70)*	3.60 *(0.69)*
All words	3.70 *(0.79)*	3.78 *(0.83)*
Range	1–6	1–6

*Note.* ^a^ Mean age-of-acquisition ratings across words and raters with the standard deviation in italics and parentheses, wherein 1 = 0–1 years, 2 = 2–3 years, 3 = 4–5 years, 4 = 6–8 years, 5 = 9–10 years, and 6 = 10 + years

**Fig. 1:**
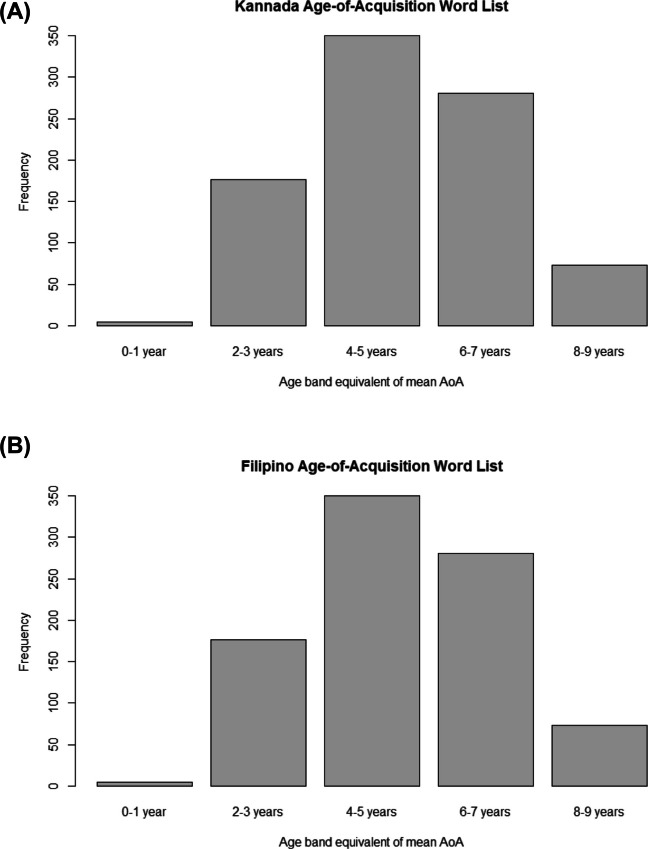
Frequency distribution of mean age-of-acquisition ratings in Kannada (**1A**) and Filipino (**1B**)

Figure 1 highlights notable crosslinguistic similarities in the frequency distribution of mean AoA ratings when converted into their equivalent age bands. The largest proportion of words in both lists was rated as acquired between 4 and 5 years (Kannada: 42%, Filipino: 40%), followed by 6–7 years (32% in both languages) and 2–3 years (20% in both). Fewer words were rated as acquired between 9 and 10 years (5% in Kannada, 8% in Filipino) and less than .01% of words in both lists were rated between 0 and 1 years. None received an average rating of age 10 +.

Table 4 contains a list of ten words with the earliest AoA ratings in the two word lists. In both languages, the list was exclusively (Filipino) or predominantly (8 of 10 Kannada words) nouns.

**Table 4:** Ten words with the earliest age-of-acquisition ratings in the Filipino and Kannada word lists

	Kannada				Filipino			
Rank	Word	Meaning	POS	Mean AoA	Word	Meaning	POS	Mean AoA
1	**haalu**	**milk**	**noun**	**1.69**	**gatas**	**milk**	**noun**	**1.88**
2	**ammanu**	**mother**	**noun**	**1.69**	**nanay**	**mother**	**noun**	**1.93**
3	**niiru**	**water**	**noun**	**1.76**	**mata**	**eye**	**noun**	**1.95**
4	**kaNNu**	**eye**	**noun**	**1.77**	kamay	hand	noun	1.97
5	**appanu**	**father**	**noun**	**1.84**	bibig	mouth	noun	1.98
6	bekku	cat	noun	1.85	**tatay**	**father**	**noun**	**2.00**
7	tinnu	eat	verb	1.97	ulo	head	noun	2.02
8	haNNu	fruit	noun	2.03	**tubig**	**water**	**noun**	**2.05**
9	hesaru	name	noun	2.04	aso	dog	noun	2.07
10	malagu	sleep	verb	2.05	ilong	nose	noun	2.07

*Notes.* Words in bold text are words with equivalent meanings in the two languages. Notably, the ten earliest rated words had equivalents in the 885-word list for the other language, except the Filipino list did not have equivalents for bekku (cat) and haNNu (fruit)

### Interrater and test–retest reliability

Interrater reliability was assessed using intra-class correlations (ICC) among participants in each sample, as well as the test–retest reliability of 50 words that were rated during the practice set and again during the main survey. Following guidelines by Koo and Li (2016), ICCs were computed using a two-way random consistency model in the R package *irr,* which assumes two random effects for raters and ratings, independent ratings between individual raters, and correlations in ratings provided by individual raters. ICC values were interpreted as follows: poor (< 0.50), moderate (0.50–0.75), good (0.75–0.90), and excellent (> 0.90).

Both samples demonstrated excellent interrater reliability: Kannada ICC =.98 (95% CI =.98–.98) and Filipino ICC =.99 (95% CI =.98–.99). Test–retest reliability ICCs, computed using the 50 words that featured in both practice and main word lists in each language, were in the moderate range: in Kannada ICC =.65 (95% CI =.63 –.66) and in Filipino ICC =.70 (95% CI =.68–.72).

We then examined the role of session timing on the test–retest reliability. Same-day testing had some impact, as ICCs were lower among Filipino participants who completed all four sessions within the same day (ICC =.66, 95% CI =.62 –.69) compared to those who completed them over multiple days (ICC =.72, 95% CI =.70 –.74). Nevertheless, test–retest ICCs were in the moderate range for both groups. This comparison was not conducted in the Kannada sample, as only one participant completed the survey on the same day.

### The role of word and rater properties in AoA ratings

Next, we examined the role of word-level and rater-level properties on AoA ratings in the two languages by running mixed-effect models on the R package *lme4*. Typical statistical approaches in AoA research, and psycholinguistics in general, involve correlation and regression-type analyses (including ANOVA) of aggregated data points across participants or items (e.g., Kuperman et al., 2012; Łuniewska et al., 2019). However, research designs that involve the same set of participants answering the same set of items yield a hierarchical data structure with responses that are correlated with one another; hence, linear mixed-effects models (LMMs) have become increasingly popular as an analytic method that could reflect this data structure.

Following the best-practice guidance outlined by Meteyard and Davies (2020), a random-effects structure was first specified. This was followed by single-predictor mixed-effects models that analyzed the relationship of each rater-level and word-level characteristics with AoA ratings, while accounting for the differences between raters (who each rated 885 items) and items (which each received a rating from every rater).

Nested models were compared to assess improvements in model fit using likelihood-ratio tests. The final models were estimated using restricted maximum likelihood (REML) methods. “I don’t know” responses were removed. Education level (1 = primary level to 4 = postgraduate level) and print age band (1 = 3 to 5 years to 3 = 9 to 10 years) were recoded into numeric predictors, with higher values representing higher levels of education and later print age bands, respectively. All numeric predictors (number of syllables, phonemes, and morphemes, print age band, education level, age, and number of languages spoken) were centered prior to data analysis. With the categorical predictors, reference categories were set for participant type (expert vs. parent or teacher), gender (female vs. male), and parts-of-speech category (noun vs. verb, adjective, adverb, or pronoun) to enable estimates between the levels of these variables. Dummy codes were assigned by *lme4*.

#### Random effects structure

First, the random-effects structure was determined by comparing random-effects models that included raters only (Model 1a), words only (Model 1b), and raters and words (Model 1c). The final random-effects only model included intercepts for both raters and words, which provided the best fit for the data and accounted for over half of the variation in AoA ratings. Detailed model comparison results are reported in the Supplementary Materials Tables S1 to S5.

#### Single-predictor models

Second, single-predictor models (Models 2a–2e and 3a–3e, described in more detail below) were tested to estimate the relationship between each word- and rater-level variable and AoA ratings, over and above the random effects of words and raters. To foreshadow the key findings, model comparison results demonstrated that each word-level property made a small but significant contribution to AoA ratings compared to the final random-effects only model, unlike the rater-level properties.

The fixed-effects estimates for all single-predictor models are reported in Table 5. In Kannada, verbs, adjectives, and adverbs had later AoA ratings compared to nouns; that is, they were generally rated as later-acquired than nouns. In contrast, on average, pronouns were rated as earlier-acquired than nouns. All other word-level properties were positively associated with Kannada AoA ratings. The more morphemes, syllables, and phonemes in a word, and the later the print age band from which a word first occurred in the corpus, the later the AoA rating. In contrast to Kannada, the analysis of Filipino data revealed that only verbs were rated as later-acquired compared to nouns; whereas, all other parts of speech were rated as acquired at a similar average age as nouns. Similar to Kannada, all of the other word-level characteristics were positively associated with AoA ratings.

**Table 5:** Fixed effect coefficients for single-predictor models in Kannada and Filipino

Variable	Estimate	SE	95% CI	*t*	*p*	Estimate	SE	95% CI	*t*	*p*
The random effects structure of Models 2a–3e included intercepts for words and raters (Model 1c in Tables S1 and S2), which provided a better fit to the data over a random effects structure that included raters only (Model 1a) and words only (Model 1b, Tables S1 and S2). See the Supplemental Materials for model comparison statistics										
	Kannada models					Filipino models				
*Word-level variables*										
Model 2a: POS										
POS [verb]^a^	0.29	0.06	0.17–0.40	4.88	** <.001**	0.14	0.07	0.01–0.26	2.09	**.036**
POS [adjective]^a^	0.27	0.10	0.08–0.46	2.81	**.005**	0.17	0.09	– 0.01–0.35	1.89	.059
POS [adverb]^a^	0.46	0.10	0.27–0.65	4.75	** <.001**	– 0.10	0.13	– 0.36–0.16	– 0.78	.435
POS [pronoun]^a^	– 0.21	0.10	– 0.40 to – 0.02	– 2.12	**.034**	– 0.10	0.11	– 0.31–0.10	– 0.97	.333
Model 2b: Number of syllables	0.36	0.02	0.31–0.41	15.20	** <.001**	0.28	0.03	0.17–0.25	11.00	** <.001**
Model 2c: Number of phonemes	0.37	0.02	0.33–0.42	16.10	** <.001**	0.33	0.03	0.28–0.38	13.00	** <.001**
Model 2d: Number of morphemes	0.19	0.03	0.14–0.24	7.66	** <.001**	0.20	0.03	0.14–0.25	7.11	**.001**
Model 2e: Print age band	0.29	0.02	0.24–0.34	12.00	** <.001**	0.36	0.03	0.31–0.41	14.40	** <.001**
*Rater-level variables*										
Model 3a: Participant type Participant type [parent]^b^	0.02	0.21	– 0.39–0.4 3	0.09	0.932	0.45	0.25	– 0.04–0.93	1.78	.079
Participant type [teacher]^b^	0.27	0.19	– 0.11–0.65	1.41	0.162	0.22	0.27	– 0.32–0.75	0.79	.433
Model 3b: Education level	– 0.08	0.09	– 0.27–0.10	– 0.87	0.386	– 0.21	0.08	– 0.04 to – 0.05	– 2.59	**.010**
Model 3c: Age	– 0.09	0.08	– 0.26–0.07	– 1.09	0.277	0.10	0.09	– 0.07–0.27	1.14	.260
Model 3d: Gender [male]^c^	0.10	0.22	– 0.33–0.52	0.44	0.660	– 0.43	0.27	– 0.95–0.09	– 1.61	.112
Model 3e: Number of languages spoken	0.09	0.08	– 0.07–0.26	1.10	0.274	– 0.20	0.14	– 0.47–0.08	– 1.40	.166

^a^Reference category = nouns. ^b^Reference category = expert. ^c^Reference category = female. *p* values in bold are significant

The rater-level variables tested in the single-predictor models were participant type, education level, age, gender, and number of languages spoken. As shown in Table 5, none of these variables significantly predicted variation in AoA ratings in both samples, with one exception. In the Filipino sample, participants’ education level was negatively associated with age-of-acquisition ratings; that is, the higher the education level, the earlier the age-of-acquisition ratings given to words. This finding might be driven by participants with postgraduate degrees who generally rated words as earlier-acquired than other participants (see Figure S1 for a boxplot visualization of the Kannada and Filipino samples). Among participants with postgraduate degrees, 53% were experts, 32% were parents, and 16% were teachers; thus, the result cannot be attributed solely to membership in the expert category.

#### Concurrent effects of word- and rater-level predictors

Following reviewer feedback, we tested multiple-predictor models that considered the concurrent effects of word-level characteristics in one model, and rater-level characteristics in another model, over and above the random effects of words and raters. Variance inflation factors (VIFs) were computed, following recommended cut-offs between 5 and 10, to ensure that the significant correlations between predictors did not impact the reliability of the models.

Multiple-predictor models that included all word-level characteristics (Model 2f: POS, number of syllables, number of phonemes, number of morphemes, and age print band) yielded high VIF statistics for number of syllables and phonemes in both languages (Kannada: 9.54–10.57, Filipino: 8.11–8.76). Hence, phoneme length was chosen over syllable length as a finer-grained measure that captures the inclusion of complex syllables and morphophonemic changes across morpheme boundaries. The resulting multiple-predictor models (Model 2g: POS, number of phonemes, number of morphemes, and age print band) demonstrated improved model fit in both languages (see Table S3).

Table 6 reports the fixed-effects estimates for the multiple-predictor models. When the concurrent effects of word-level characteristics were considered, word length (number of phonemes) and age print bands were positively associated with Kannada and Filipino AoA ratings, similar to the single-predictor model results. In contrast, the number of morphemes was no longer associated with AoA ratings in both languages when POS type, number of phonemes, and print age band were held constant. Controlling for other word characteristics also changed some associations between POS types and AoA ratings in the two languages. Earlier significant associations for Kannada pronouns and Filipino verbs were no longer present. Kannada verbs, adjectives, and adverbs were still rated as later-acquired compared to nouns. Filipino adjectives, which had non-significant associations with AoA ratings in the single-predictor models, were rated as later-acquired compared to nouns in the multiple-predictor models. This suggests that some of the differences in AoA across PoS found in the single-predictor model may be attributable to the other factors now taken into account.

**Table 6:** Fixed effect coefficients for multiple-predictor models in Kannada and Filipino (Model 2g)

Variable	Estimate	SE	95% CI	*t*	*p*	Estimate	SE	95% CI	*t*	*p*
The random effects structure of Model 2g included intercepts for words and raters (Model 1c), which provided a better fit to the data over a random effects structure that included raters only (Model 1a) and words only (Model 1b). See the Supplemental Materials for model comparison statistics										
	Kannada models					Filipino models				
*Model 2g: Word-level variables*										
POS										
POS [verb]^a^	0.25	0.19	0.15–0.35	5.03	** <.001**	0.02	0.01	0.11–0.15	0.28	.780
POS [adjective]^a^	0.31	0.23	0.16–0.47	3.90	** <.001**	0.25	0.18	0.10–0.40	3.24	**.001**
POS [adverb]^a^	0.35	0.26	0.19–0.51	4.28	** <.001**	– 0.02	– 0.02	– 0.24–0.19	– 0.21	.832
POS [pronoun]^a^	– 0.02	– 0.02	– 0.18–0.14	– 0.29	.771	– 0.08	0.06	– 0.10–0.26	0.82	.411
Number of phonemes	0.33	0.24	0.20–0.28	11.39	** <.001**	0.32	0.24	0.26–0.39	9.57	** <.001**
Number of morphemes	– 0.03	– 0.02	– 0.08–0.03	– 0.92	.356	– 0.05	– 0.04	– 0.13–0.03	– 1.30	.192
Print age band	0.24	0.18	0.20–0.29	11.19	** <.001**	0.32	0.23	0.27–0.37	13.58	** <.001**

^a^ Reference category = nouns. *p* values in bold are significant

In contrast, likelihood-ratio tests indicated that the multiple-predictor models for rater characteristics (Model 3f) did not significantly improve the explanation of variation in AoA ratings in either language (see Table S4); hence, no fixed effects estimates for these models are reported.

#### Number of morphemes and word length

Results of the multiple-predictor models suggest that the effects of number of morphemes on AoA ratings are attenuated when other word properties are taken into account. However, the theoretical interpretability of these results could be hampered by the confound between word length (measured in the number of phonemes) and number of morphemes, which are highly correlated in the two agglutinative languages in this study (Kannada *r* =.63; Filipino *r* =.70).

We explored a focused approach to identify the potential unique contribution of the number of morphemes to the AoA ratings, over and above word length. We purposively selected words with the same number of phonemes but varying numbers of morphemes and re-estimated the relationship between the number of morphemes and AoA, over and above the random effects of words and raters. Words with nine phonemes were selected as this length contained an optimal distribution of one to four morpheme words (see Supplemental Materials Figure S2 and Figure S3), reducing the word lists to 115 Kannada and 103 Filipino words with all five POS types included.

We tested single-predictor models that examined the effect of the number of morphemes in the length-restricted word list, over and above the random effects of words and raters (Model 4). The number of morphemes had weak but significant associations with AoA ratings. Whereas this association was positive in Filipino, the association was negative in Kannada (see Table 7 for fixed-effect estimates, with model comparison results reported in Table S5). Whereas the analyses demonstrate that the number of morphemes can predict AoA ratings among words that are identical in phoneme length, mixed results regarding the direction of this relationship suggest the impact of third variables that were not considered in this analysis. We turn to this issue in the Discussion.

**Table 7:** Fixed effect coefficients for the length-restricted model (Model 4) in Kannada and Filipino

Variable	Estimate	SE	95% CI	*t*	*p*	Estimate	SE	95% CI	*t*	*p*
The random effects structure of Model 4 included intercepts for words and raters (Model 1c), which provided a better fit to the data over a random effects structure that included raters only (Model 1a) and words only (Model 1b). Models 1a–1c were re-estimated on the restricted word list. See the Supplemental Materials for model comparison statistics										
	Kannada model 4					Filipino model 4				
Number of morphemes	– 0.19	0.06	– 0.31 to – 0.07	– 3.18	**0.002**	0.15	0.07	0.01–0.28	2.04	.**044**

*Note. p*-values in bold are significant

#### Print age band and other word-level properties

Finally, we conducted a chi-square analysis to examine the consistency of age-band classifications based on age-of-acquisition ratings and first occurrence in print. This analysis serves as a validity check given the absence of other AoA norms, child language assessments, or other behavioral measures that can be used as reference data in either language.

**Fig. 2:**
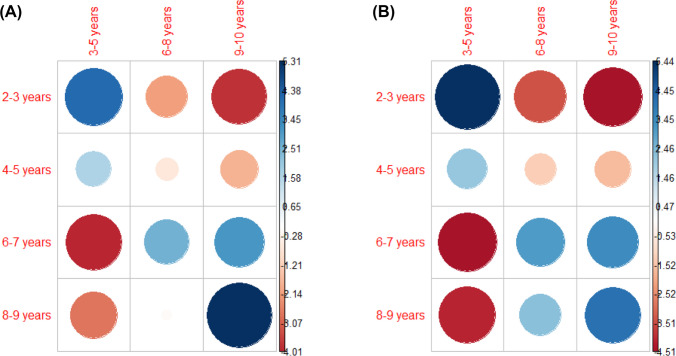
Chi-square residual plots for Kannada (**2a**) and Filipino (**2b**) illustrating the relationship between AoA age band classifications (*rows*) and print age band (*columns*). Positive residuals, which indicate a positive association between row and column values, are indicated in *blue*, whereas negative residuals (indicating a negative association) are indicated in *red*. The size and color saturation of the circle are proportional to the amount of contribution to the chi-square score

Given that expected values for cells within the 0–1 year AoA age band were less than five in both languages, the tests were conducted by analyzing the distribution of words across four AoA age bands (2–3, 4–5, 6–7, 8–9) and the three print age bands (3–5, 6–8, 9–10). Chi-square results in both languages revealed a significant relationship between the two variables, *X*^2^ (6, *N* = 875) = 112, *p* <.001 (Kannada), *X*^2^ (6, *N* = 880) = 151, *p* <.001 (Filipino). Figure 2a and b illustrate the associations between rows and columns of the contingency table, highlighting the points of attraction (positive associations) and repulsion (negative associations) between cells. The relative contributions of each cell to the chi-square score were also computed as percentages to further illustrate the nature of the dependency.

In both languages, words that were rated as acquired at 2–3 years were positively associated with words that first occurred in books for 3–5 year olds (15% contribution to the Chi-square score in Kannada; 19% in Filipino) and negatively associated with books for older children (Kannada: 4–13% contribution, Filipino: 8–13% contribution). Similar but more modest trends could be seen among words with an equivalent AoA rating of 4–5 years (ranging from 0.40–3% contribution in the two languages). The trend reversed at the older AoA age bands, such that words that were rated as acquired at 6–7 and 8–9 years were negatively associated with the 3–5 print age band (Kannada: 7–14% contribution; Filipino: 11–13% contribution), weakly but generally positively associated with books for 6–8 (Kannada: 0.02%–6%; Filipino: 3–6% contribution), and positively associated with books for 9–10 year olds (Kannada: 9–25% contribution; Filipino: 8–11% contribution). In Kannada, the association was especially strong for words classified within the 8–9 AoA age band and 9–10 print age band (25% contribution). In sum, the chi-square analysis revealed a general pattern of consistency between rater-provided AoA ratings and print age band, with earlier-acquired words also appearing earlier in child-directed print, and vice versa.

## Discussion

In this study, we presented a new set of age-of-acquisition norms for two agglutinative languages, Kannada and Filipino. These norms were gathered from parents, teachers, and experts using a modified task instruction that asked when children learned a word. Beyond characterizing 885 words in each language, we found consistent trends in the two languages in the overall distribution of AoA ratings, the types of words with the earliest ratings, and the pattern of associations between AoA and word and rater level characteristics. Single-predictor models revealed that word characteristics, including parts-of-speech category, word length, number of morphemes, and age band of first occurrence in a print corpus were significantly correlated with Kannada and Filipino AoA ratings. However, analyzing the concurrent effects of word characteristics attenuated the effects of the number of morphemes and changed the patterns of relationships between parts-of-speech categories and AoA in both languages. In contrast, rater characteristics had a limited impact on AoA judgments in both single- and multiple-predictor models. Further analysis examined the relevance of morphology to AoA using a tightly controlled word list; however, mixed results suggest that multiple underlying linguistic factors may drive this relationship in the two agglutinative languages. We interpret and consider the implications of these results below.

### What do word characteristics reveal about age-of-acquisition in Kannada and Filipino?

The list of ten words with the earliest age-of-acquisition ratings in both Kannada and Filipino is dominated by nouns. Remarkably, five out of ten of the earliest-rated words in the two languages (*milk*, *mother*, *eye*, *water*, and *father*) have equivalent meanings despite no deliberate attempt to match the lists. Similarly, early adult self-rated AoA ratings were observed in English (*mama, water, eye:* Kuperman et al., 2012), Mandarin Chinese (*mother, father:* Xu et al., 2021), and across 25 languages in the Łuniewska et al. (2016) study for *eye*. Three of the words were also identified within the top 20 words in a study examining MB-CDI parent checklists, including *mother* and *father* (noted in the US, Hong Kong, and Beijing) and *milk* (noted in Hong Kong) (Tardif et al., 2008). The crosslinguistic MB-CDI database Wordbank likewise demonstrated high proportions of children producing equivalents to most of the ten words in the Kannada and Filipino lists within the first 36 months of life in different languages (e.g., English, Turkish, Cantonese; see https://wordbank.stanford.edu/). The earlier rated ten words also have some consistencies with child speech data, which have identified the Kannada words for mother (*amma*) and father (*appa*) (Bharandwaj et al., 2015) and the Filipino words for hand (*kamay*), mouth (*bibig*), and eye (*mata*) (Marzan, 2013) as frequently mentioned nouns among young children. Overall, the results appear consistent with suggestions that children learn words in similar sequences across languages (Łuniewska et al., 2016), particularly words for people and concrete nouns (Braginsky et al., 2019; Frank et al., 2017; Tardif et al., 2008).

In Kannada, verbs, adjectives, and adverbs generally attained later AoA ratings than nouns. In Filipino, only adjectives received later AoA ratings than nouns, when other factors were taken into account. The finding that the relationship between the word POS and AoA is modulated by word length and age of first exposure (as indexed by print age band) suggests that multiple factors may contribute to when words from different lexical categories are acquired (e.g., Braginsky et al., 2019). For instance, in Kannada, two verbs attracted the earliest ratings (*eat*, *sleep*), pointing to the role of frequent early input verb learning, similar to the findings for high frequency verbs, for example, *go* in English (Theakston, et al, 2002). The positive relationship between word length measures and AoA ratings, when other factors are taken into account, is consistent with the observed word length effect in adult self-rated and parent checklist-based AoA across languages, including agglutinative (e.g., Braginsky et al., 2019; Łuniewska et al., 2016).

However, caution is needed when interpreting the observed differences between POS. As noted by a reviewer, the presentation of words by POS may have induced priming effects in some sections of the rating task. The randomization applied across sessions, POS, and word blocks, as well as moderate levels of test–retest reliability observed between practice and main study lists, may have partially mitigated these effects.

Results related to the relationship between the number of morphemes and AoA revealed mixed associations, offering a first assessment of how morphology might impact AoA in agglutinative languages. When assessed in a single-predictor model, words with more morphemes tended to be rated as later-acquired than words with fewer morphemes, supporting the idea of a generally later acquisition of multimorphemic forms. Although there was no direct comparison of AoA between base forms and their multimorphemic forms, this result is consistent with findings of Davies et al. (2016), validating the usefulness of the number of morphemes as an index of morphological complexity. However, there is a clear need to go beyond a simple count of morphemes, which remains a proxy measure for linguistic processes at play and is inevitably confounded with word length measures, as demonstrated by the multiple-predictor models.

We explored an approach to isolate the unique contribution of morphology over and above word length. When the analysis is constrained to words with nine phonemes and one to four morphemes, the relationship between the number of morphemes and AoA is negative in Kannada and positive in Filipino. What these seemingly contradictory results potentially reveal is an interplay between semantics and morphosyntax; that is, AoA of morphologically complex words is likely to be driven both by the lexical meaning of the ‘base’ word, as highlighted by Kuperman et al. (2012), and the morphological information that is attached to it, as demonstrated by Davies et al. (2016). This interplay may be particularly pronounced in agglutinative languages, as the structure of the language necessitates separate morphemes that reflect both the semantic and grammatical functions of the word. The nine-phoneme word lists in both languages contained roughly similar proportions of monomorphemic words (Kannada: 14%; Filipino: 13%) but contrasted in their distribution of words with two (Kannada: 45%; Filipino: 56%) and three morphemes (Kannada: 37%; Filipino: 30%). The Kannada word list was more likely than Filipino to contain early-AoA multimorphemic words in the two- and three-morpheme range, such as *iruvegaLu* (meaning: ants, with the suffix -*gaLu* as a plural marker) and *ettikoLLu* (meaning: lift, with suffixes *-ko-* and *-LLu* to denote a reflexive sense of “taking”). Although multimorphemic, these words would likely be judged as highly concrete and contain highly frequent bases and affixes, echoing results by Braginsky et al. (2019). Such early-AoA multimorphemic words were also present in the Filipino list, such as *masugatan* (meaning: to get wounded, with the circumfix *ma- -an* to denote who got wounded), but less frequently so. The Filipino list has a relatively higher proportion of higher-AoA multimorphemic words wherein the root word is likely to be judged as more abstract and less frequent, such as *nahihibang* (meaning: delirious, with the prefix *na-* and the reduplicated first syllable to denote an ongoing state).

Additionally, the number of morphemes cannot capture the morphophonemic changes across morpheme boundaries in the two languages, which may or may not involve the insertion of additional phonemes in these words. These processes could, in part, be captured by the number of phonemes measure, which is consistently positively correlated with AoA ratings. Moreover, the number of morphemes could neither account for phonological neighborhood density effects, which were highlighted in studies looking at morphological acquisition in agglutinative languages (e.g., Engelmann et al., 2019; Granlund et al., 2019), nor other sources of morphological complexity, including polysemy, homonymy, and allomorphy (Nag, 2025). Overall, the results provide rich avenues for further study; for example, by examining what aspects of morphological complexity play a role in AoA and how they might interact. These may include the transparency of semantic or consistency of morphophonological processes present, the frequency of component morphemes, and their semantic and grammatical function.

### What can we learn from the relationship between AoA and age of occurrence in print?

The results revealed a positive association between adult ratings of children’s age-of-acquisition and age band of first occurrence in child-directed print, providing additional validation for the new AoA norms. Extending beyond this, another implication is that print exposure could be a viable means of supporting children’s word acquisition, whether through shared book reading or through independent reading by novice readers. Nevertheless, differences between words in child-directed print and speech have been noted previously: English-language children’s books are found to contain more unique word types (Montag et al., 2015) and longer and more morphologically complex words (Dawson et al., 2021, 2023) than are heard in speech. If this is the case for a morphologically simpler language such as English, then the same question presents an interesting avenue for future research in morphologically rich languages as well.

### Do rater characteristics drive variation in AoA ratings? Mostly no.

The analysis did not find any systematic variation in AoA ratings by the type of rater (parent, teacher, or expert), age, gender, or the number of languages that they speak, contributing to the sparse literature that demonstrated mixed trends across these characteristics. However, methodological differences between studies should also be acknowledged in the interpretation of these results. First, Łuniewska et al. (2016) found that parents generally provided earlier AoA ratings than non-parents, who were assumed not to have any recent experiences with children. In contrast, all participants in the current study were selected on the basis of their personal or professional experience with children, and could have provided similar ratings due to this shared experience (however, it is unclear if the teachers and experts in the study were also parents). In this case, differences in inclusion criteria could potentially account for the discrepancy between findings. Second, it was perhaps not possible to detect a potential gender difference in the current study due to the low absolute number of male participants (Kannada: 12; Filipino: 9); hence, we could not replicate Kuperman et al. (2012)’s earlier result of later AoA ratings among women in the sample nor can we claim to provide further support for Łuniewska et al. (2016)’s finding of non-significant gender differences in AoA ratings. With age and language status variables, there is no clear explanation for the mixed findings. Non-significant age associations in the current study were inconsistent with Kuperman et al. (2012)’s finding of later AoA among older participants but consistent with Łuniewska et al. (2016)’s non-significant findings. Finally, Łuniewska et al. (2016) found that bi-/multi-linguals provided later AoA ratings than monolinguals; whereas, language status was not significantly related to AoA ratings in this study. Given that non-significant results do not necessarily translate to an absence of an effect, all that can be concluded is that we did not detect any differences in AoA due to participant type, age, gender, and language status in these samples, and that there is no evidence to suggest that these rater characteristics impact our earlier interpretations regarding the links between word characteristics and AoA.

However, Filipino participants’ educational attainment was inversely associated with age-of-acquisition ratings in the single-predictor model; that is, the higher the education level, the earlier the AoA ratings. Do participants with higher education backgrounds tend to observe earlier AoA among the children they interact with, or do they overestimate the time course of children’s language development? It is difficult to support either interpretation based on the available data. Comparing adults’ AoA ratings against direct measures of children’s word acquisition patterns could potentially clarify the impact of education level on AoA estimates, at least in the Filipino context.

### Methodological considerations and future directions

The study is characterized by a sample that was purposively chosen based on their experience with typically developing children, which diverges from samples recruited from undergraduate populations or from general adult populations. Similarly, the core question to elicit subjective AoA ratings diverged from the classic Carroll and White (1973) instruction, which focused on raters’ own acquisition experience, and instead focused on child-focused ratings. Our approach presents a quick and resource-efficient method to assess aspects of child language development and has the advantage of providing norms for a wider age range than MB-CDI and other parental checklist measures. This could be an appealing solution, especially for researchers of understudied languages, wherein documentation on AoA and child language acquisition is relatively sparse.

While the general consistency between AoA and age of occurrence in a print corpus provides an initial indication of validity for the new word lists, further validation of this approach to AoA measurement should echo the approach taken by Smolík and Filip (2022). First is to measure the correlations between the new AoA norms and both early and school-age vocabulary assessments to assess how well parent, teacher, and expert reports map onto the sequence of word acquisition. Second is to assess the relationship between AoA norms and adult word naming and lexical decision results, to examine their utility in adult psycholinguistics research.

## Conclusion

The present study introduces the age-of-acquisition ratings of 885 words in Kannada and Filipino, two agglutinative languages that are underrepresented in AoA and child language research. The words represent five different parts-of-speech categories and are tagged for word length, number of morphemes, and age band of first occurrence in child-directed print. The analyses suggest that the ratings are internally reliable and robust, and are generally consistent with the sequence of occurrence in print and patterns of acquisition demonstrated in other languages. Despite differences in linguistic features and word selection, Kannada and Filipino show similarities in age-of-acquisition trends. Notably, results suggest potential impacts of morphology on AoA ratings and its interactions with other word characteristics, and enable further investigations of the underlying drivers of morphological complexity. Given that the AoA rating task has been tailored to focus on child language acquisition and administered to participants with direct experience of having or working with children, these lists are especially useful for users who are searching for words acquired in the preschool and early primary levels and can be used in future research, as well as the development of assessment, teaching, and intervention materials for speakers of these languages.

## Supplementary Information

Below is the link to the electronic supplementary material.

Supplementary file1 (DOCX 215 KB)

## Data Availability

The technical reports for the age-of-acquisition study and book leveling tool, age-of-acquisition word lists, and datasets are available at the TalkTogether Open Science Framework repository (10.17605/OSF.IO/3ZDFN).

## Footnotes

1 Filipino is often used interchangeably with Tagalog, a regional language spoken in Metro Manila and its surrounding regions. 76 million is the estimate of L1 and L2 Tagalog speakers in the Philippines, which is understood as the total number of Filipino speakers in the country.

## Code availability

The analysis code for data analysis is available at 10.17605/OSF.IO/3ZDFN.

## Declarations

### Open practices statement

The study did not undergo pre-registration.
