# Supplementary material for: Measurement of age-of-acquisition in morphologically rich languages: Insights from Kannada and Filipino
Source: Behav Res Methods. 2025 Dec 1;58(1):11. doi: 10.3758/s13428-025-02876-z (PMC12669312; doi:10.3758/s13428-025-02876-z)
Supplement: Supplementary file 1 — Supplementary file1 (DOCX 215 KB) [file 13428_2025_2876_MOESM1_ESM.docx]

Supplemental Materials

Mixed-Effects Model Building Procedure

In both languages, a simple random-effects model was estimated with random intercepts for raters (model 1a), words (model 1b), and raters and words (model 1c), as illustrated in Tables S1 (Kannada) and S2 (Filipino). Model comparisons showed that the random effects structure should account for both rater- and word-level intercepts in AoA ratings in the Kannada and Filipino samples, as shown in the comparison between models 1a and 1b, and 1c respectively. Conditional R^2^ values for model 1c suggest that over half of the variation in AoA ratings can be attributed to raters and words (random effects), given that this model contains no fixed effects.

Subsequent single-predictor mixed-effects models (single-predictor: models 2a – 3e) were constructed by including fixed effects for each rater-level and word-level variable separately, and including the random effects structure as specified in model 1c. The marginal R^2^ values for these models describe the proportion of variance explained by each word- and rater-level variable (fixed effects) whereas the conditional R^2^ values describe the variance explained by both fixed and random effects. The fixed effect coefficients associated with each model are presented in the main text.

As shown in Table S1 and Table S2, model comparisons and marginal R^2^ estimates demonstrated that each of the measured word-level properties (parts-of-speech, number of syllables, phonemes, morphemes, and print age band) had a weak but significant contribution to the variation in the AoA ratings, over and above the random effects included in the model.

A similar approach of entering fixed effects on top of a random effects structure that include intercepts for raters and words was used for the multiple-predictor models (Models 2g and 3f) and the restricted-length models (Model 4).

Table S1. Mixed-Effects Model Comparisons for Single-Predictor Models in Kannada (Models 1a-3e)

| Sampling Units | N total obs = 64,805 (“I don’t know” responses and responses for three non-corpus words removed)  N raters = 74; N items = 882 | | | | | | | | | |  |  |
| --- | --- | --- | --- | --- | --- | --- | --- | --- | --- | --- | --- | --- |
| Model number | Nested model | Fixed effects added | Random effects | | Model fit | | | | LRT test against nested | | R^2^ | |
|  |  |  | Raters | Words | AIC | BIC | LL | df | df | X^2^ | Marginal | Conditional |
| *1. Determining the random effects structure* | | | | | | | | | | |  |  |
| 1a | - | - | Intercepts | - | 203585 | 203612 | -101789 | 3 | - | - | 0 | .253 |
| 1b | - | - | - | Intercepts | 200351 | 200378 | -100173 | 3 | - | - | 0 | .318 |
| 1c | 1a; 1b | - | Intercepts | Intercepts | 170643 | 170679 | -85317 | 4 | 1 | 1a vs. 1c: 32944.00***  1b vs. 1c: 29710.00*** | 0 | .577 |
| *2. Fixed effects of word-level variables (single predictor models)* | | | | | | | | | | |  |  |
| 2a | 1c | Parts-of-speech | Intercepts | Intercepts | 170598 | 170670 | -85291 | 8 | 4 | 53.00*** | .019 | .578 |
| 2b | 1c | Number of syllables | Intercepts | Intercepts | 170439 | 170485 | -85215 | 5 | 1 | 205.55*** | .068 | .577 |
| 2c | 1c | Number of phonemes | Intercepts | Intercepts | 170418 | 170463 | -85204 | 5 | 1 | 227.10*** | .074 | .577 |
| 2d | 1c | Number of morphemes | Intercepts | Intercepts | 170588 | 170633 | -85289 | 5 | 1 | 56.80*** | .020 | .577 |
| 2e | 1c | Print age band | Intercepts | Intercepts | 170511 | 170556 | -85250 | 5 | 1 | 134.04*** | .046 | .577 |
| *3. Fixed effects of rater-level variables (single predictor models)* | | | | | | | | | | |  |  |
| 3a | 1c | Participant type | Intercepts | Intercepts | 170644 | 170698 | -85316 | 6 | 2 | 2.00 | .009 | .580 |
| 3b | 1c | Education level | Intercepts | Intercepts | 170644 | 170689 | -85317 | 5 | 1 | 0.78 | .003 | .579 |
| 3c | 1c | Age | Intercepts | Intercepts | 170644 | 170689 | -85317 | 5 | 1 | 1.22 | .004 | .579 |
| 3d | 1c | Gender | Intercepts | Intercepts | 170645 | 170690 | -85317 | 5 | 1 | 0.20 | .001 | .579 |
| 3e | 1c | Number of languages spoken | Intercepts | Intercepts | 170644 | 170689 | -85317 | 5 | 1 | 1.24 | .004 | .579 |
|  |  |  |  |  |  |  |  |  |  |  |  |  |

*Notes.* * *p* < .05, ** *p <* .01, *** *p <* .001. The marginal R^2^ represents the proportion of variance explained only by the fixed effects; whereas, the conditional R^2^ represents the proportion of variance explained by the fixed and random effects together.

Table S2. Mixed-Effects Model Comparisons for Single-Predictor Models in Filipino (Models 1a-3e)

| Sampling Units | N total obs = 59,495 (“I don’t know” responses removed)  N raters = 68; N items = 885 | | | | | | | | | |  |  |
| --- | --- | --- | --- | --- | --- | --- | --- | --- | --- | --- | --- | --- |
| Model number | Nested model | Fixed effects added | Random effects | | Model fit | | | | LRT test against nested | | R^2^ | |
|  |  |  | Raters | Words | AIC | BIC | LL | df | df | X^2^ | Marginal | Conditional |
| *1. Determining the random effects structure* | | | | | | | | | | |  |  |
| 1a | - | - | Intercepts | - | 186756 | 186783 | -93375 | 3 | - | - | 0 | .292 |
| 1b | - | - | - | Intercepts | 184782 | 184809 | -92388 | 3 | - | - | 0 | .345 |
| 1c | 1a; 1b | - | Intercepts | Intercepts | 149477 | 149513 | -74734 | 4 | 1 | 1a vs. 1c: 37282.00***  1b vs. 1c: 35307.00*** | 0 | .650 |
| *2. Fixed effects of word-level variables* | | | | | | | | | | |  |  |
| 2a | 1c | Parts-of-speech | Intercepts | Intercepts | 149474 | 149546 | -74729 | 8 | 4 | 10.65* | .004 | .650 |
| 2b | 1c | Number of syllables | Intercepts | Intercepts | 149366 | 149411 | -74678 | 5 | 1 | 112.89*** | .043 | .650 |
| 2c | 1c | Number of phonemes | Intercepts | Intercepts | 149323 | 149368 | -74656 | 5 | 1 | 155.72*** | .058 | .650 |
| 2d | 1c | Number of morphemes | Intercepts | Intercepts | 149429 | 149474 | -74710 | 5 | 1 | 49.24*** | .019 | .650 |
| 2e | 1c | Print age band | Intercepts | Intercepts | 149291 | 149336 | -74641 | 5 | 1 | 187.27*** | .068 | .650 |
| *3. Fixed effects of rater-level variables* | | | | | | | | | | |  |  |
| 3a | 1c | Participant type | Intercepts | Intercepts | 149477 | 149531 | -74733 | 6 | 2 | 3.60 | .015 | .653 |
| 3b | 1c | Education level | Intercepts | Intercepts | 149472 | 149517 | -74731 | 5 | 1 | 6.57* | .027 | .651 |
| 3c | 1c | Age | Intercepts | Intercepts | 149477 | 149522 | -74734 | 5 | 1 | 1.32 | .006 | .651 |
| 3d | 1c | Gender | Intercepts | Intercepts | 149476 | 149521 | -74733 | 5 | 1 | 2.61 | .011 | .651 |
| 3e | 1c | Number of languages spoken | Intercepts | Intercepts | 149477 | 149522 | -74733 | 5 | 1 | 1.99 | .008 | .651 |
|  |  |  |  |  |  |  |  |  |  |  |  |  |

*Notes.* * *p* < .05, ** *p <* .01, *** *p <* .001. The marginal R^2^ represents the proportion of variance explained only by the fixed effects; whereas, the conditional R^2^ represents the proportion of variance explained by the fixed and random effects together.


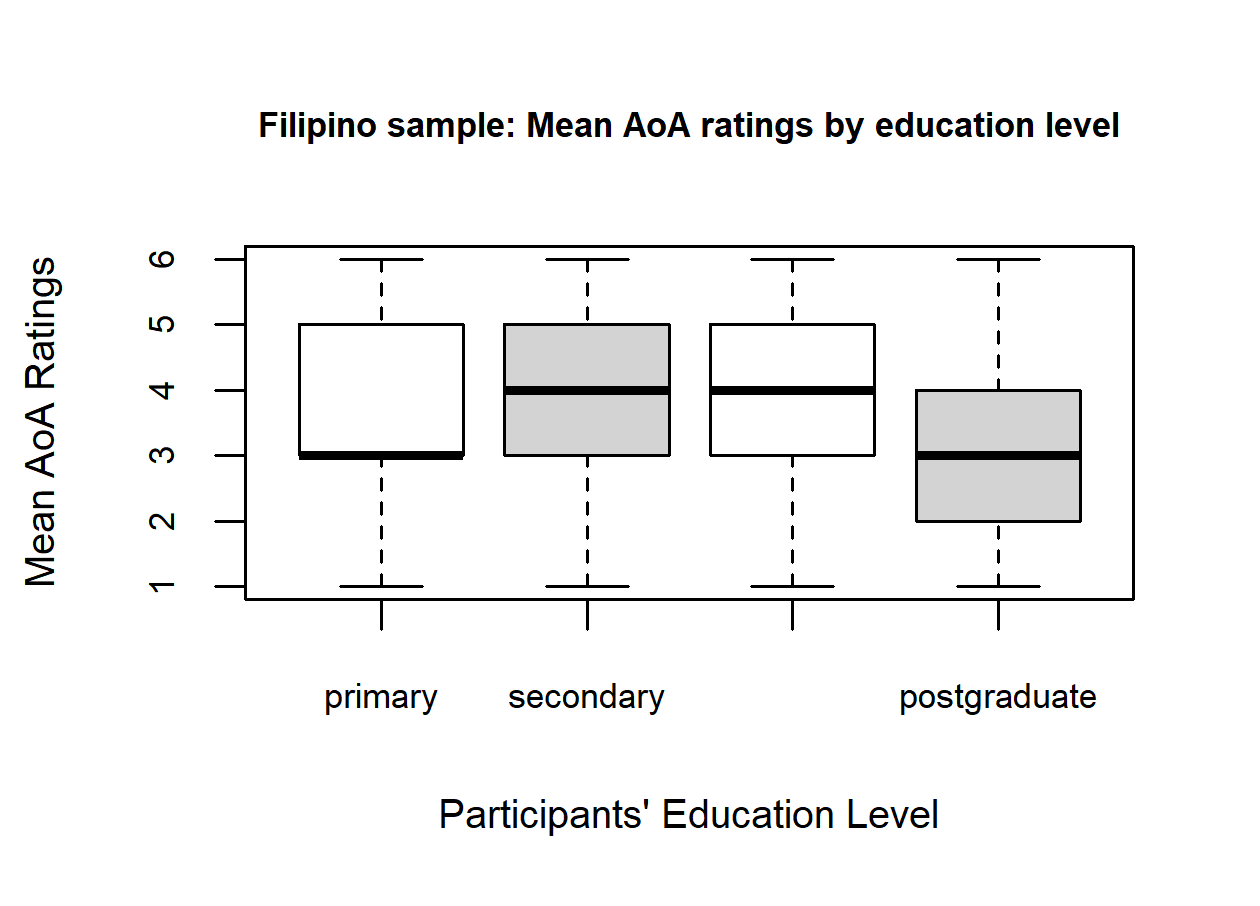

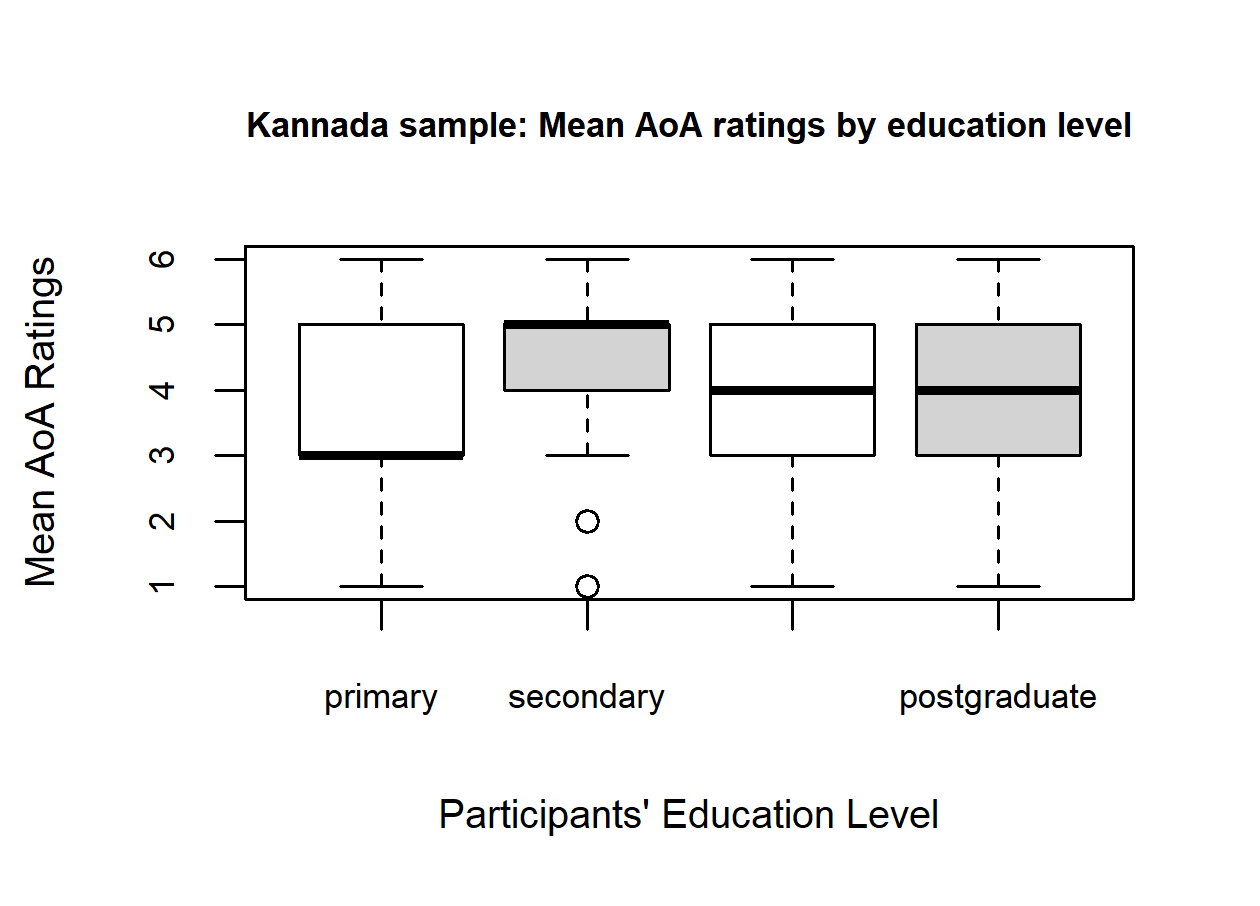


**(S1A)**

**(S1B)**

Figure S1. Mean age-of-acquisition ratings by rater education level (Kannada: S1a; Filipino: S1b)

Table S3. Mixed-Effects Model Comparisons for the Multiple-Predictor Model of Word Characteristics in Kannada and Filipino (Model 2g)

| Model number | Nested model | | Fixed effects added | Random effects | | Model fit | | | | LRT test against nested | | R^2^ | |
| --- | --- | --- | --- | --- | --- | --- | --- | --- | --- | --- | --- | --- | --- |
|  |  | |  | Raters | Words | AIC | BIC | LL | df | df | X^2^ | Marginal | Conditional |
| Kannada | | | | | | | | | | | | | |
| *2. Fixed effects of word-level variables (multiple predictor model)* | | | | | | | | | | | | | |
| 2g | 1c | | Parts-of-speech; number of phonemes; number of morphemes; age print band | Intercepts | Intercepts | 170273 | 170373 | -85126 | 11 | 7 | 331.00*** | .115 | .578 |
| Filipino | | | | | | | | | | | | | |
|  | |  | | | | | | | | | | | |
| *2. Fixed effects of word-level variables (multiple predictor model)* | | | | | | | | | | | | | |
| 2g | 1c | | Parts-of-speech; number of phonemes; number of morphemes; age print band | Intercepts | Intercepts | 149150 | 149249 | -74564 | 11 | 7 | 341.00*** | .114 | .650 |
|  |  | |  |  |  |  |  |  |  |  |  |  |  |

*Notes.* * *p* < .05, ** *p <* .01, *** *p <* .001. The marginal R^2^ represents the proportion of variance explained only by the fixed effects; whereas, the conditional R^2^ represents the proportion of variance explained by the fixed and random effects together. Model 2f included the number of syllables; however, VIF statistics between number of syllables and phonemes exceeded recommended cut-offs of 5-10. Number of syllables was dropped in Model 2g, prioritizing number of phonemes as a finer-grained measure of word length. All LRT tests between Model 2g and single-predictor models 2a-2e with are also significant at *p <* .001 in both languages.

Table S4. Mixed-Effects Model Comparisons for the Multiple-Predictor Model of Rater Characteristics in Kannada and Filipino (Model 3f)

| Model number | Nested model | | Fixed effects added | Random effects | | Model fit | | | | LRT test against nested | | R^2^ | |
| --- | --- | --- | --- | --- | --- | --- | --- | --- | --- | --- | --- | --- | --- |
|  |  | |  | Raters | Words | AIC | BIC | LL | df | df | X^2^ | Marginal | Conditional |
| Kannada | | | | | | | | | | | | | |
| *3. Fixed effects of rater-level variables (multiple predictor model)* | | | | | | | | | | | | | |
| 3f | 1c | | Participant type;  education level; age;  gender; number of languages spoken | Intercepts | Intercepts | 170644 | 170735 | -85312 | 10 | 6 | 10.8 | .034 | .585 |
|  |  | |  |  |  |  |  |  |  |  |  |  |  |
| Filipino | | | | | | | | | | | | | |
|  | |  | | | | | | | | | | | |
| *3. Fixed effects of rater-level variables (multiple predictor model)* | | | | | | | | | | | | | |
| 3f | 1c | | Participant type;  education level; age;  gender; number of languages spoken | Intercepts | Intercepts | 149478 | 149568 | -74729 | 10 | 6 | 10.8 | .042 | .658 |
|  |  | |  |  |  |  |  |  |  |  |  |  |  |
|  |  | |  |  |  |  |  |  |  |  |  |  |  |

*Notes.* * *p* < .05, ** *p <* .01, *** *p <* .001. The marginal R^2^ represents the proportion of variance explained only by the fixed effects; whereas, the conditional R^2^ represents the proportion of variance explained by the fixed and random effects together. Because model comparison results were not significant, fixed-effects coefficients are not reported in this manuscript. The VIF in both languages (Kannada: 1.14-1.72; Filipino: 1.25-2.08) was below recommended cut-offs between 5-10.


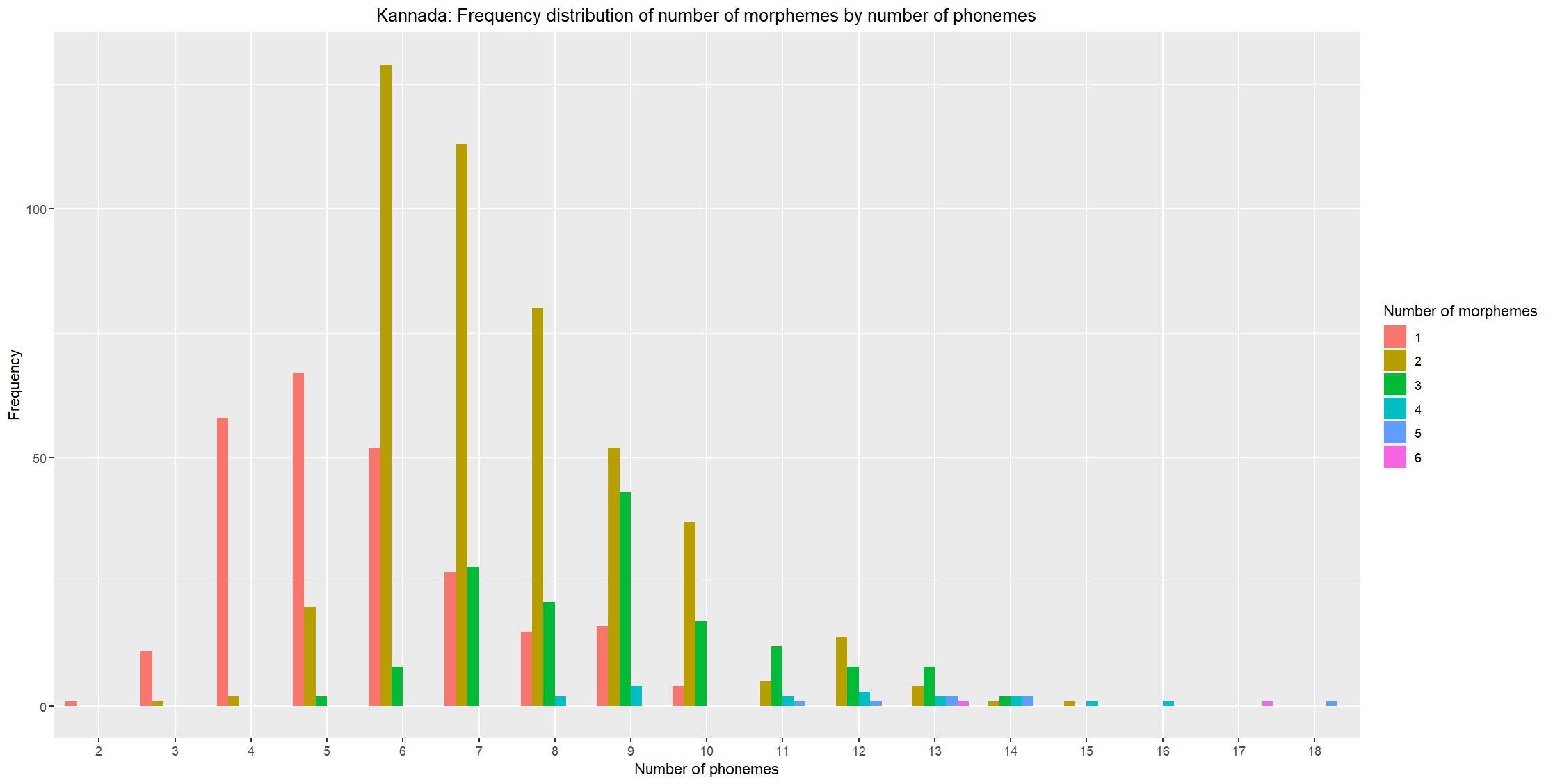


Figure S2. Frequency distribution of 885 words by number of morphemes and number of phonemes in Kannada


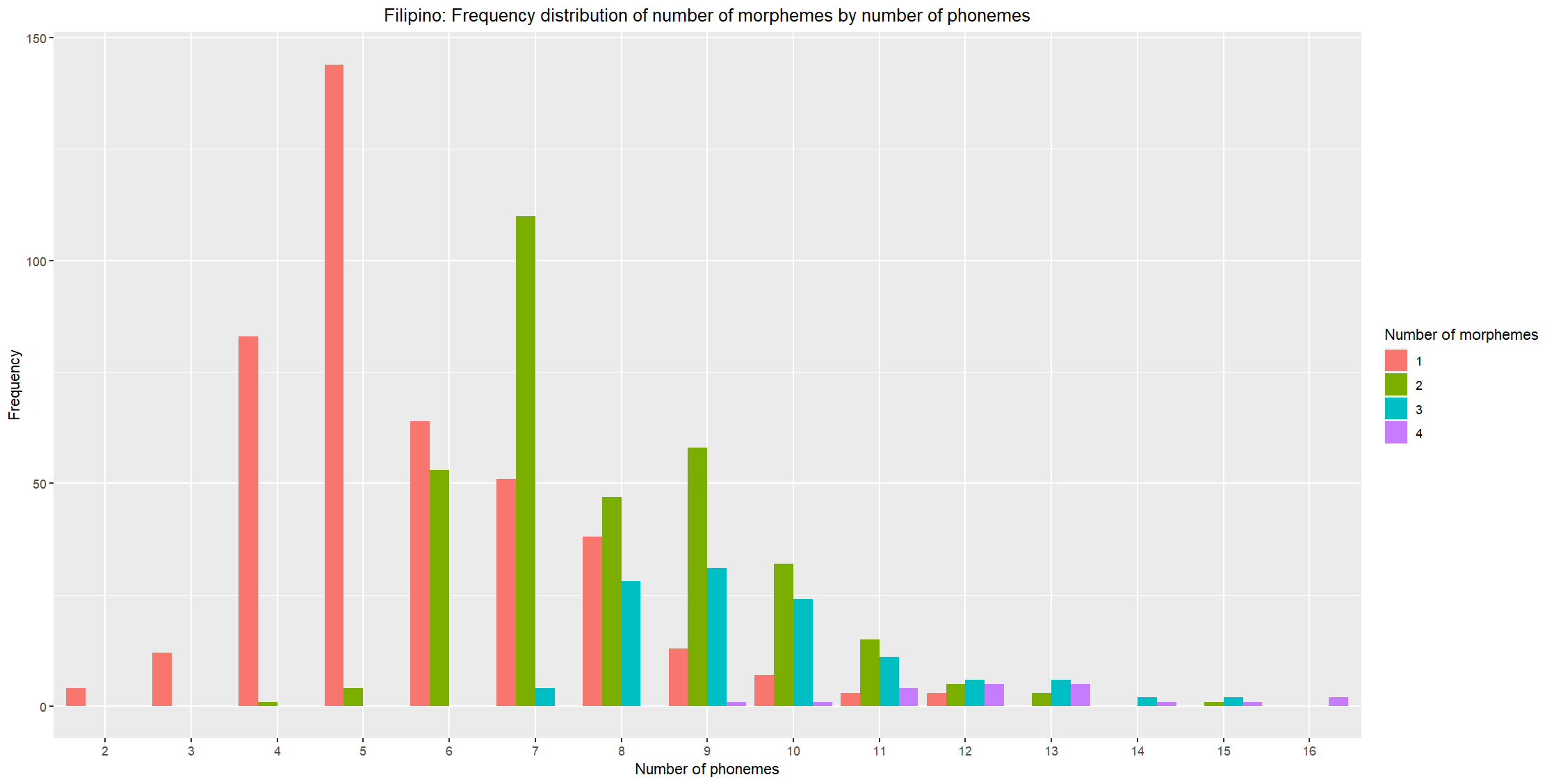


Figure S3. Frequency distribution of 885 words by number of morphemes and number of phonemes in Filipino

Table S5. Mixed-Effects Model Comparisons for the Length-Restricted Model in Kannada and Filipino (Model 4)

| Model number | Nested model | | Fixed effects added | Random effects | | Model fit | | | | LRT test against nested | | R^2^ | |
| --- | --- | --- | --- | --- | --- | --- | --- | --- | --- | --- | --- | --- | --- |
|  |  | |  | Raters | Words | AIC | BIC | LL | df | df | X^2^ | Marginal | Conditional |
| Kannada | | | | | | | | | | | | | |
| *1. Determining the random effects structure*^a^ | | | | | | | | | | | | | |
| 1a | - | | - | Intercepts | - | 25485 | 25506 | -12740 | 3 | - | - | 0 | .284 |
| 1b | - | | - | - | Intercepts | 25876 | 25897 | -12935 | 3 | - | - | 0 | .258 |
| 1c | 1a; 1b | | - | Intercepts | Intercepts | 22110 | 22138 | -11051 | 4 | 1 | 1a vs. 1c: 3377.20***  1b vs. 1c: 3768.40*** | 0 | .549 |
|  |  | |  |  |  |  |  |  |  |  |  |  |  |
| *4. Fixed effect of number of morphemes on the AoA of words with 9 phonemes*^a^ | | | | | | | | | | | | | |
| 4 | 1c | | Number of morphemes | Intercepts | Intercepts | 22102 | 22137 | -11046 | 5 | 1 | 9.80** | .022 | .550 |
| Filipino | | | | | | | | | | | | | |
|  | |  | | | | | | | | | | | |
| *1. Determining the random effects structure*^b^ | | | | | | | | | | | | | |
| 1a | - | | - | Intercepts | - | 21068 | 21088 | -10531 | 3 | - | - | 0 | .325 |
| 1b | - | | - | - | Intercepts | 21509 | 21529 | -10751 | 3 | - | - | 0 | .285 |
| 1c | 1a; 1b | | - | Intercepts | Intercepts | 17150 | 17538 | -8751 | 4 | 1 | 1a vs. 1c: 3559.60***  1b vs. 1c: 4000.50*** | 0 | .628 |
|  |  | |  |  |  |  |  |  |  |  |  |  |  |
| *4. Fixed effect of number of morphemes on the AoA of words with 9 phonemes*^b^ | | | | | | | | | | | | |  |
| 4 | 1c^a^ | | Number of morphemes | Intercepts | Intercepts | 17508 | 17542 | -8749 | 5 | 1 | 4.14* | .012 | .629 |
|  |  | |  |  |  |  |  |  |  |  |  |  |  |

*Notes.* * *p* < .05, ** *p <* .01, *** *p <* .001. ^a^ Sampling units for Kannada Model 4: N total observations = 8414; N items = 115 ^b^ Sampling units for Filipino Model 4: N total observations = 6900; N items = 103. The marginal R^2^ represents the proportion of variance explained only by the fixed effects; whereas, the conditional R^2^ represents the proportion of variance explained by the fixed and random effects together. Random effects models 1a-1c were re-estimated with the restricted word list to enable model comparisons.
